# The influence of chest X-ray results on antibiotic prescription for childhood pneumonia in the emergency department

**DOI:** 10.1007/s00431-021-03996-2

**Published:** 2021-03-22

**Authors:** Josephine S. van de Maat, Daniella Garcia Perez, Gertjan J. A. Driessen, Anne-Marie van Wermeskerken, Frank J. Smit, Jeroen G. Noordzij, Gerdien Tramper-Stranders, Charlie C. Obihara, Jeanine Punt, Henriette A. Moll, Rianne Oostenbrink

**Affiliations:** 1 Department of General Paediatrics, Erasmus MC – Sophia Children’s Hospital, P.O. Box 2060, 3000 CB Rotterdam, The Netherlands; 2 Department of Paediatrics, HAGA-Juliana Children’s Hospital, Den Haag, The Netherlands; 3 Department of Paediatrics, Flevoziekenhuis, Almere, The Netherlands; 4 Department of Paediatrics, Maasstad Ziekenhuis, Rotterdam, The Netherlands; 5 Department of Paediatrics, Reinier de Graaf Gasthuis, Delft, The Netherlands; 6 Department of Paediatrics, Franciscus Gasthuis &Vlietland, locatie Gasthuis, Rotterdam, The Netherlands; 7 Department of Paediatrics, Elisabeth Tweestedenziekenhuis, Tilburg, The Netherlands; 8 Department of Paediatrics, Langeland Ziekenhuis, Zoetermeer, The Netherlands

**Keywords:** Paediatrics, Emergency medical services, Pneumonia, Diagnostic techniques and procedures, Guideline adherence, anti-bacterial agents

## Abstract

The aim of this study is to evaluate the influence of chest X-ray (CXR) results on antibiotic prescription in children suspected of lower respiratory tract infections (RTI) in the emergency department (ED). We performed a secondary analysis of a stepped-wedge, cluster randomized trial of children aged 1 month to 5 years with fever and cough/dyspnoea in 8 EDs in the Netherlands (2016–2018), including a 1-week follow-up. We analysed the observational data of the pre-intervention period, using multivariable logistic regression to evaluate the influence of CXR result on antibiotic prescription. We included 597 children (median age 17 months [IQR 9–30, 61% male). CXR was performed in 109/597 (18%) of children (range across hospitals 9 to 50%); 52/109 (48%) showed focal infiltrates. Children who underwent CXR were more likely to receive antibiotics, also when adjusted for clinical signs and symptoms, hospital and CXR result (OR 7.25 [95% CI 2.48–21.2]). Abnormalities on CXR were not significantly associated with antibiotic prescription.

*Conclusion*: Performance of CXR was independently associated with more antibiotic prescription, regardless of its results. The limited influence of CXR results on antibiotic prescription highlights the inferior role of CXR on treatment decisions for suspected lower RTI in the ED.

**Table:** 

**What is Known:** *• Chest X-ray (CXR) has a high inter-observer variability and cannot distinguish between bacterial or viral pneumonia.* *• Current guidelines recommend against routine use of CXR in children with uncomplicated respiratory tract infections (RTIs) in the outpatient setting.*	
**What is New:** *• CXR is still frequently performed in non-complex children suspected of lower RTIs in the emergency department* *• CXR performance was independently associated with more antibiotic prescriptions, regardless of its results, highlighting the inferior role of chest X-rays in treatment decisions.*

**Supplementary Information:**

The online version contains supplementary material available at 10.1007/s00431-021-03996-2.

## Introduction

Community-acquired pneumonia (CAP) is one of the leading causes of childhood morbidity and mortality worldwide. Although in Western countries mortality has significantly declined, CAP continues to cause a high burden of disease [1]. Pneumonia is a common reason for children to visit the emergency department (ED) and contributes to substantial use of medical services, including hospitalization, emergency care visits and antibiotic use [2, 3].

Chest radiography (CXR) was long considered the reference standard for diagnosing CAP in children with suspected lower respiratory tract infections (RTI). However, more recent evidence shows the limitations of CXR in guiding the management of these children, like the high inter-observer variability, inability to distinguish viral from bacterial pneumonia and radiation exposure [4–7]. Inter-observer variability of CXR reading for paediatric pneumonia has shown to be present between radiologists as well as between various other specialists [8–10]. Reported reasons for this are lack of radiological training of treating physicians, lack of clinical information available for radiologists and human error. In 2011, guidelines for the management of childhood CAP were published in Europe and the USA [5, 6], recommending against routine use of CXR in most children in the outpatient setting, and restricting the use of CXR to children with moderate to severe signs and symptoms of CAP at risk of developing complications.

Some studies have evaluated the impact of the CAP guidelines on diagnosis and treatment of childhood CAP, and did not find significant changes in CXR performance rates [11–14]. However, their study populations were limited to children with a confirmed diagnosis of CAP rather than those with signs and symptoms of a lower RTI, and they did not evaluate the impact of CXR results on antibiotic treatment. Little is known on how the CXR is currently used in antibiotic treatment decisions in the broad population of children with signs and symptoms of a lower RTI in the paediatric ED.

This study is a secondary analysis of a stepped-wedge, cluster randomized trial that evaluated the impact of a clinical decision rule on antibiotic prescription in children under 5 years of age with a suspected lower RTI in the ED (STRAP trial) [15]. In the current study, we used the pre-intervention (usual care) data of this trial to evaluate the influence of CXR results on antibiotic prescription in children with suspected lower RTI in the ED.

## Materials and methods

### Study design

We used usual care data from the Study to Reduce Antibiotic prescription in childhood Pneumonia (STRAP, Netherlands Trial Register, NTR5326) [15]. STRAP is a stepped-wedge cluster randomized trial, implementing a validated clinical prediction model (the Feverkidstool) [16] in the EDs of eight hospitals in the Netherlands. In this secondary analysis, we only used data from the pre-intervention period, when usual care was provided. During usual care, the patients were first triaged and assessed by a nurse. Then they were evaluated by a physician, who decided on additional diagnostics and treatment. Usual care was provided according to the Dutch guideline for febrile children [17], which is in line with the international CAP guidelines of the British Thoracic Society and the Infectious Diseases Society of America, including the recommendation to not routinely perform a CXR in the outpatient setting [5, 6]. Detailed methods of the trial have been published earlier [15].

### Population

We included children aged 1 month to 5 years presenting to the ED with fever (≥38.5 °C or reported by parents) and symptoms of a lower RTI (cough, dyspnoea or tachypnea) from January 1, 2016, to March 11, 2018. Exclusion criteria were comorbidities (immunodeficiency, multiple handicaps, congenital heart defects, chronic pulmonary disease, or preterm birth <32 weeks and aged <1 year old at the time of ED visit), use of antibiotics in the week prior to inclusion, amoxicillin allergy, another identifiable infectious focus other than lower respiratory (e.g. cutaneous, otitis, tonsillitis), and signs of complicated lower RTI at presentation (saturation <85%, respiratory insufficiency, empyema, sepsis).

### Endpoints

The endpoint for this study was antibiotic prescription (yes/no) at the end of the ED visit.

### Data collection and definitions

Data were obtained using a standardized case record form completed during the ED visit and during telephone follow-up 7 days after the ED visit. We collected data on patient’s general characteristics, clinical signs and symptoms, diagnostic tests, discharge diagnosis, treatment and strategy failure. Discharge diagnosis was determined by the treating physician at the time of ED evaluation. We used the following predefined definition of strategy failure that was used in the trial: secondary hospitalization or secondary or switched antibiotic prescription during follow-up, oxygen need or fever at day 7 or the development of complications (parapneumonic effusion, pleura-empyema, lung abscess, respiratory insufficiency).

The CXR results were defined based on the routine report of the radiologist in the electronic patient record. CXR results were classified as focal infiltrate if the report included “infiltrate”, “consolidation” or “pneumonia”. Reports including “atelectasis”, “diffuse abnormality” and “perihilar abnormality” were classified as diffuse or perihilar abnormalities. If “pleural effusion” or “empyema” was reported, the CXR was classified as “pleural effusion”. If the CXR report included the terms “normal chest”, “no abnormalities” or “clear lungs”, this was considered a normal CXR result [18].

### Statistical analyses

We used logistic regression to test the influence of the performance and results of a CXR on antibiotic prescription, adjusted for clinical signs and symptoms and hospital variability. We could include 17 predictors in our multivariable model. Next to “hospital” and “CXR result”, we included the following clinical predictors in the model: age, sex, ill appearance, hypoxia (oxygen saturation <94%), tachypnea, retractions (as a marker of increased work of breathing) and C-reactive protein (CRP) level, which are known predictors for bacterial pneumonia, based on the literature and guidelines [5, 6, 16]. Missing predictor variables were imputed 10 times using the mice package in R (version 3.3.2) [19]. The imputation model included relevant information about clinical signs and symptoms, diagnostic work-up and outcome, treatment and follow-up. Analyses were performed on all 10 databases and the results were pooled. We used IBM SPSS Statistics version 24 and R (version 4.0.0) for data management and analyses.

### Ethics

The Erasmus MC Medical Ethics Committee granted ethical approval for the STRAP study (MEC-2014-332), and written informed consent was obtained from all participants.

## Results

### Baseline characteristics

We included a total of 597 children, with a median age 17 months (IQR 9–30), and 364/597 (61%) were male (Table 1 and Online Resource 1). Ill appearance was present in 220/572 (38%) and hypoxia in 144/595 (24%) of children, and median CRP level was 19 mg/L (7–44). Antibiotics were prescribed in 179/597 (30%) of the children, and 329/597 (55%) of the children were hospitalized. The majority of children improved within a week after ED visit, but strategy failure was observed in 131/597 (22%), most frequently due to secondary antibiotic prescription or fever at day 7.

**Table 1:** Baseline characteristics

	*n* (%)	Antibiotic prescription^a^
General characteristics		
Hospital		
Hospital A	69/597 (12%)	11/69 (16%)
Hospital B	35/597 (6%)	8/35 (23%)
Hospital C	144/597 (24%)	40/144 (28%)
Hospital D	123/597 (21%)	33/123 (27%)
Hospital E	82/597 (14%)	30/82 (37%)
Hospital F	95/597 (16%)	42/95 (44%)
Hospital G	29/597 (5%)	11/29 (38%)
Hospital H	20/597 (3%)	4/20 (20%)
Clinical characteristics		
Male sex	364/597 (61%)	
Age in years, median (IQR)	17 (9–30)	
Ill appearance	220/572 (38%)	
Oxygen saturation <94%	144/595 (24%)	
Chest X-ray		
Chest X-ray result		
Normal	26/597 (4%)	
Focal infiltrate/consolidation	52/597 (9%)	
Diffuse/perihilar abnormality	31/597 (5%)	
Therapy and follow-up		
Antibiotic prescription	179/597 (30%)	
Hospitalization	329/597 (55%)	
Strategy failure	131/597 (22%)	
Strategy failure, reasons:		
Secondary antibiotic prescription	45/597 (8%)	
Changed antibiotic prescription during follow-up^b^	14/597 (2%)	
Secondary hospitalization	16/597 (3%)	
Oxygen need at day 7	9/597 (2%)	
Fever at day 7	47/597 (8%)	

^a^Given the small numbers, differences between hospitals should be interpreted with caution

^b^Including one ICU admission

### Chest X-ray use

In 109/597 (18%) of the population, a CXR was performed. This varied across hospitals from 11/123 (9%, 95% CI 4–14%) to 10/20 (50%, 95% CI 28–72%). Of the 109 obtained CXRs, 52 (48%) showed focal infiltrates, 31 (28%) showed diffuse or perihilar findings, and 26 (24%) showed no abnormalities. None of the CXRs showed pleural effusion.

### Influence of chest X-ray performance and result on antibiotic prescription

**Fig. 1:**
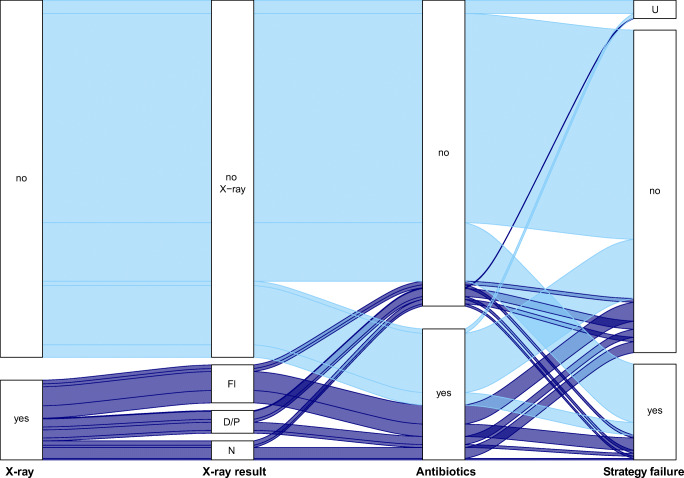
Flow of patients from ED visit to follow-up, starting from the decision to perform a chest X-ray (yes/no) to strategy failure (yes/no/unknown) 7 days after the ED visit. Light blue, no chest X-ray; dark blue, chest X-ray performed. FI, focal infiltrate; D/P, diffuse/perihilar findings; N, normal; U, unknown. Numbers underlying this figure can be found in Online Resource 2

Figure 1 shows the flow of children from ED presentation to 7 days after the ED, including the performance and results of the CXR, antibiotic prescription and strategy failure. Of the 52 children with a focal infiltrate on the CXR, all but nine received antibiotics. Four of these nine untreated children had strategy failure during follow-up (all had secondary antibiotic prescription). Strategy failure was higher in children who underwent a CXR (34/108, 31%) than in those who did not (97/464, 21%). More than half (32/57, 56%) of the children with diffuse/perihilar or no abnormalities on their CXR received antibiotic treatment. Of all children that underwent CXR, 69% (75/109) received antibiotics, versus 21% (104/488) of children that did not undergo CXR.

When we adjusted for hospital variability, clinical signs and symptoms and result of the CXR in a multivariable analysis, we found that the mere performance of a CXR was independently associated with antibiotic prescription (OR 7.25 [95% CI 2.48–21.2]); see Table 2. Older age, CRP level and ill appearance were other predictors for antibiotic prescription. Abnormalities on CXR (focal or diffuse/perihilar abnormalities) showed higher odds ratios (Table 2), but were not significantly associated with antibiotic prescription.

**Table 2:** Influence of CXR performance and result on antibiotic prescription

	Unadjusted OR (95% CI)	Adjusted OR^a^ (95% CI)
Chest X-ray performed (yes)	8.09 (5.11–12.8)	7.25 (2.48–21.2)
Chest X-ray result		
Normal	Reference	Reference
Focal infiltrate	2.53 (0.86–7.46)	1.88 (0.48–7.32)
Diffuse/perihilar abnormalities	0.5 (0.17–1.45)	0.32 (0.08–1.29)

^a^Model is adjusted for hospital, age, sex, ill appearance, tachypnea, hypoxia, retractions, and CRP level. The full model can be found in Online Resource 3

## Discussion

### Main results

In a multicentre population of children under 5 years presenting with a suspected lower RTI in eight paediatric EDs, a CXR was performed in 18%. Almost half of these CXRs showed focal infiltrates, and a quarter showed diffuse or perihilar findings. The decision to perform a CXR as part of the diagnostic work-up was associated with more frequent antibiotic prescription. This association remained after correcting for hospital variation, clinical signs and symptoms and result of the CXR. Results of the CXR, as presence of focal or diffuse abnormalities, were not significantly associated with antibiotic prescription.

### Interpretation and comparison with previous studies

The high number of abnormalities on performed CXRs in our population suggests that physicians ordering them already had a high clinical suspicion of CAP and that their clinical judgements were generally accurate. We observed variability in CXR use across hospitals, which has been reported previously, although not always at individual patient level [11, 20, 21]. The observed variability in CXR use across participating hospitals in our study is similar to previous findings from studies with similar inclusion criteria based on respiratory symptoms, showing CXR performance rates between 9 and 36% [20, 22, 23].

It is striking that the decision to perform a CXR was independently associated with antibiotic treatment, but the results of the CXR were not. Nearly half of children with normal CXRs still received antibiotics. Similar to our results, previous studies have shown that a CXR does not result in changes in management [4, 7] and that antibiotic prescription decisions depend on the physician’s intention to treat, regardless of the CXR result [24]. Previous studies have also shown that children who undergo CXR are more likely to receive antibiotics, despite low numbers of diagnosed pneumonia [22, 25]. Other factors, like clinical assessment, appear to be more important than CXR results in the decision to prescribe antibiotics.

The current guidelines recommend to not routinely perform a CXR in case of non-complicated CAP [5, 6]. The children in our population mostly had uncomplicated disease at presentation, given the fact that none of the CXRs showed pleural effusion or empyema. This is also reflected by a relative low proportion of ill appearance. In contrast, the majority of children were classified as at least “urgent” during triage. Triage, however, may be more related to a high proportion of children with either dyspnoea, hypoxia or high fever, which all may result in higher urgency at triage. Ill appearance, as a proxy of clinician Gestalt, is more a general assessment of overall illness. Strategy failure was present in 22% of children, but it must be noted that this was using a broad trial definition [15], including signs of a prolonged disease course like fever at day 7. So, in our non-complex population (without comorbidities or prior antibiotic treatment), the chances of detecting a complicated pneumonia on CXR are very low, confirming the guideline recommendations.

### Strengths and limitations

To the best of our knowledge, this is the first European study that evaluated CXR use in children with suspected CAP in the ED after the publication of the international guidelines for the management of childhood CAP (British and US guidelines published in 2011, Dutch guideline in 2013) [5, 6, 17]. Strengths of our study include its prospective and multicentre design and well-defined, broad study population. We included children with signs and symptoms of lower RTIs rather than children diagnosed with CAP, reflecting more accurately the population of children presenting to the ED.

The results of this study should be interpreted in the light of the following limitations. First, the population is limited to a trial population. Even though we used the pre-intervention data only, the use of the trial’s strict exclusion criteria may have affected the generalizability of our results to the complete ED population. Second, we adjusted for clinical signs and symptoms in our regression model, but we did not have information on the exact considerations of the physicians to order a CXR or not. Last, we did not consider the inter-observer variability between radiologists and paediatricians in our analyses. For our analysis we intentionally used the radiologist’s reading exclusively, because this was most consistently available. We collected data on the radiologist’s as well as the paediatrician’s CXR readings and found a kappa of 0.59 for agreement (i.e. moderate agreement), which is similar to previous studies [9, 26, 27]. The high inter-observer variability is a well-recognized limitation of CXR [28].

### Implications

Our results show that there is a very limited role of the CXR in the diagnostic and therapeutic pathway of childhood CAP in ED settings. In line with the current guidelines, performance of a CXR in non-complex children suspected of a lower RTI should be discouraged. In the absence of a gold standard for CAP, we need other tools to support the physician’s decisions on diagnostics and treatment. Clinical decision rules based on individual risk prediction of bacterial infections may be used for this purpose [15, 16, 29, 30]. Other upcoming diagnostic techniques for diagnosing childhood CAP are point of care lung ultrasound and new point of care biomarkers [31]. Further improvement of these new techniques is necessary to support the physician’s decisions.

## Conclusion

One-third of children suspected of lower RTIs receive antibiotics in the ED, and CXR is still frequently performed in a non-complex population. CXR use was associated with more antibiotic prescriptions, regardless of the CXR results. The limited influence of CXR results on antibiotic prescription highlights the inferior role of CXR in treatment decisions. Our findings support the guideline recommendations against routine use of CXR for children with uncomplicated CAP. Further research should aim to identify new diagnostic techniques in order to optimize the management of childhood pneumonia.

## Supplementary information

ESM 1 (PDF 28 kb)

ESM 2 (DOCX 25 kb)

## Data Availability

Individual participant data that underlie the results reported in this article will be made available after de-identification at time of article publication, ending 10 years following article publication. Data will be shared with investigators who provide a methodologically sound proposal, designed to achieve aims in the approved proposal, or for individual participant data meta-analysis. Data are deposited in the repository of Data Archiving and Networked Services (DANS, doi: 10.17026/dans-27a-fj4k). Proposals should be directed to info@dans.knaw.nl; to gain access, data requestors will need to sign a data access agreement.

## Abbreviations

CAP: Community-acquired pneumonia
CRP: C-reactive protein
CXR: Chest X-ray
ED: Emergency department
ICU: Intensive care unit
IQR: Interquartile range
OR: Odds ratio
RTI: Respiratory tract infection
STRAP: Study To Reduce Antibiotic prescription in childhood Pneumonia

## Code availability

Code will be shared with investigators who request this from the corresponding author.
