# Supplementary material for: The influence of chest X-ray results on antibiotic prescription for childhood pneumonia in the emergency department
Source: Eur J Pediatr. 2021 Mar 22;180(9):2765–72. doi: 10.1007/s00431-021-03996-2 (PMC8346381; doi:10.1007/s00431-021-03996-2)
Supplement: Supplementary file 2 — (DOCX 25 kb) [file 431_2021_3996_MOESM2_ESM.docx]

**Supplementary information**

**Table 1. Extended baseline information**

| **General characteristics** | n (%) |
| --- | --- |
| Hospital |  |
| Hospital A | 69/597 (12%) |
| Hospital B | 35/597 (6%) |
| Hospital C | 144/597 (24%) |
| Hospital D | 123/597 (21%) |
| Hospital E | 82/597 (14%) |
| Hospital F | 95/597 (16%) |
| Hospital G | 29/597 (5%) |
| Hospital H | 20/597 (3%) |
| Triage level |  |
| Immediate | 13/506 (3%) |
| Very urgent | 293/506 (58%) |
| Urgent | 146/506 (29%) |
| Standard or non-urgent | 54/506 (11%) |
| **Clinical characteristics** |  |
| Male sex | 364/597 (61%) |
| Age in years, median (IQR) | 17 (9-30) |
| Duration of fever in days, median (IQR) | 2 (1-4) |
| Ill appearance | 220/572 (38%) |
| Cough | 555/581 (96%) |
| Dyspnea | 432/581 (74%) |
| Oxygen saturation <94% | 144/595 (24%) |
| **Diagnostic work-up** |  |
| C-reactive protein test done | 375/597 (63%) |
| C-reactive protein, median (IQR) | 19 (7-44) |
| Chest X-ray result |  |
| Normal | 26/597 (4%) |
| Focal infiltrate/consolidation | 52/597 (9%) |
| Diffuse/perihilar abnormality | 31/597 (5%) |
| Discharge diagnosis |  |
| Pneumonia | 204/594 (34%) |
| Bronchiolitis | 117/594 (20%) |
| Upper RTI | 176/594 (30%) |
| Viral induced wheeze | 69/594 (12%) |
| Subglottic laryngitis | 21/594 (4%) |
| Other | 7/594 (1%) |
| **Therapy and follow-up** |  |
| Antibiotic prescription | 179/597 (30%) |
| Hospitalization | 329/597 (55%) |
| Strategy failure | 131/597 (22%) |
| Strategy failure, reasons: | 0/597 (0%) |
| Secondary antibiotic prescription | 45/597 (8%) |
| Changed antibiotic prescription during follow-up^a^ | 14/597 (2%) |
| Secondary hospitalization | 16/597 (3%) |
| Oxygen need at day 7 | 9/597 (2%) |
| Fever at day 7 | 47/597 (8%) |

*Footnote: ^a^ including one ICU-admission*

**Table 2. Numbers underlying flow diagram of Figure 1.**

| **Xray** | **Xray result** | **Antibiotics** | **Strategy failure** | **Frequency** |
| --- | --- | --- | --- | --- |
| yes | focal infiltrate | yes | yes | **8** |
| yes | focal infiltrate | yes | no | **35** |
| yes | focal infiltrate | no | yes | **4** |
| yes | focal infiltrate | no | no | **5** |
| yes | diffuse abnormalities | yes | yes | **4** |
| yes | diffuse abnormalities | yes | no | **11** |
| yes | diffuse abnormalities | no | yes | **2** |
| yes | diffuse abnormalities | no | no | **13** |
| yes | diffuse abnormalities | no | unknown | **1** |
| yes | normal | yes | yes | **0** |
| yes | normal | yes | no | **17** |
| yes | normal | no | yes | **3** |
| yes | normal | no | no | **6** |
| no | no x-ray | yes | yes | **7** |
| no | no x-ray | yes | no | **91** |
| no | no x-ray | yes | unknown | **6** |
| no | no x-ray | no | yes | **47** |
| no | no x-ray | no | no | **319** |
| no | no x-ray | no | unknown | **18** |
|  |  |  |  | **total n = 597** |

**Table 3. Influence of CXR performance and result on antibiotic prescription, full model**

|  | **Unadjusted OR (95% CI)** | **Adjusted OR (95% CI)** |
| --- | --- | --- |
| Hospital |  |  |
| Hospital H | reference | reference |
| Hospital A | 0.76 (0.21-2.7) | 0.89 (0.16-5.12) |
| Hospital B | 1.19 (0.31-4.57) | 0.58 (0.08-4.01) |
| Hospital C | 1.54 (0.48-4.88) | 2.92 (0.65-13.19) |
| Hospital D | 1.47 (0.46-4.71) | 2.29 (0.49-10.81) |
| Hospital E | 2.31 (0.71-7.54) | 2.14 (0.43-10.68) |
| Hospital F | 3.19 (0.99-10.23) | 2.92 (0.6-14.12) |
| Hospital G | 2.44 (0.65-9.22) | 5.59 (0.99-31.63) |
| Age (months) | **1.04 (1.02-1.05)** | **1.02 (1.01-1.04)** |
| Gender (female) | 1.09 (0.77-1.56) | 1.12 (0.67-1.85) |
| Ill appearance | **4.14 (2.84-6.04)** | **2.68 (1.54-4.68)** |
| Tachypnea | **1.83 (1.07-3.11)** | 1.31 (0.61-2.82) |
| Hypoxia (<94%) | 1.33 (0.89-1.98) | 0.77 (0.43-1.4) |
| Retractions | 1.14 (0.78-1.66) | 1.26 (0.72-2.23) |
| CRP (mg/L) | **1.04 (1.03-1.05)** | **1.04 (1.03-1.05)** |
| Chest X-ray performed (yes) | **8.09 (5.11-12.8)** | **7.25 (2.48-21.2)** |
| Chest X-ray result |  |  |
| Normal | reference | reference |
| Focal infiltrate | 2.53 (0.86-7.46) | 1.88 (0.48-7.32) |
| Diffuse / perihilar abnormalities | 0.5 (0.17-1.45) | 0.32 (0.08-1.29) |
